# Regional Brain Gray Matter Changes in Patients with Type 2 Diabetes Mellitus

**DOI:** 10.1038/s41598-020-67022-5

**Published:** 2020-06-18

**Authors:** Bhaswati Roy, Luke Ehlert, Rashmi Mullur, Matthew J. Freeby, Mary A. Woo, Rajesh Kumar, Sarah Choi

**Affiliations:** 1 Department of Anesthesiology, University of California Los Angeles, Los Angeles, CA 90095 USA; 2 Department of Medicine, University of California Los Angeles, Los Angeles, CA 90095 USA; 3 UCLA School of Nursing, University of California Los Angeles, Los Angeles, CA 90095 USA; 4 Department of Radiology, University of California Los Angeles, Los Angeles, CA 90095 USA; 5 Department of Bioengineering, University of California Los Angeles, Los Angeles, CA 90095 USA; 6 Brain Research Institute, University of California Los Angeles, Los Angeles, CA 90095 USA

**Keywords:** Health care, Medical imaging, Magnetic resonance imaging

## Abstract

Patients with Type 2 diabetes mellitus (T2DM) show cognitive and mood impairment, indicating potential for brain injury in regions that control these functions. However, brain tissue integrity in cognition, anxiety, and depression regulatory sites, and their associations with these functional deficits in T2DM subjects remain unclear. We examined gray matter (GM) changes in 34 T2DM and 88 control subjects using high-resolution T1-weighted images, collected from a 3.0-Tesla magnetic resonance imaging scanner, and assessed anxiety [Beck Anxiety Inventory], depressive symptoms [Beck Depression Inventory-II], and cognition [Montreal Cognitive Assessment]. We also investigated relationships between GM status of cognitive and mood control sites and these scores in T2DM. Significantly increased anxiety (p = 0.003) and depression (p = 0.001), and reduced cognition (p = 0.002) appeared in T2DM over controls. Decreased GM volumes appeared in several regions in T2DM patients, including the prefrontal, hippocampus, amygdala, insular, cingulate, cerebellum, caudate, basal-forebrain, and thalamus areas (p < 0.01). GM volumes were significantly associated with anxiety (r = −0.456,p = 0.009), depression (r = −0.465,p = 0.01), and cognition (r = 0.455,p = 0.009) scores in regions associated with those regulations (prefrontal cortices, hippocampus, para hippocampus, amygdala, insula, cingulate, caudate, thalamus, and cerebellum) in T2DM patients. Patients with T2DM show brain damage in regions that are involved in cognition, anxiety, and depression control, and these tissue alterations are associated with functional deficits. The findings indicate that mood and cognitive deficits in T2DM patients has brain structural basis in the condition.

## Introduction

An estimated 500 million adults worldwide live with Type 2 diabetes mellitus (T2DM), which accounts for more than 90% of all diabetes cases^1^. Prevalence and increasing mortality rates are comparable between high- and low-income countries (53.8–103.1per 1000)^2^. Additionally, people living with T2DM have a nearly twofold higher risk of neuropsychological dysfunctions, than individuals without a diabetes diagnosis^3^. Such functional deficits indicate a possibility of underlying brain pathology.

Previous T2DM studies examining brain tissue have reported cortical and subcortical atrophy, symptomatic or asymptomatic infarcts, and association with white matter lesions in periventricular and subcortical areas^4,5^. Voxel-based morphometry (VBM), an automatic quantitative volumetric technique, allows whole-brain voxel-wise comparisons of gray matter volume between groups. Although the VBM procedures have been used previously to examine gray matter changes in T2DM patients^6–8^, multiple studies are inconsistent showing tissue changes in key brain areas, including the hippocampus and other gray and white matter sites^9–12^, and further studies are needed to examine if specific brain sites have damage in T2DM. Meta-analyses revealed significant global reductions in total brain volume, orbitofrontal cortex, hippocampus, basal ganglia^13^, but significant differences in frontal and temporal volumes, anterior cingulate, superior temporal, and parietal regions, as shown in other studies^6,7,14–17^, are inconsistent.

Depression, anxiety, and cognitive impairment have been associated with T2DM^18,19^. The prevalence of depression is nearly twice that of non-diabetics^20^, and elevated anxiety symptoms are reported in up to 40% of those living with T2DM^21^. Depression and anxiety exhibit hypothalamic-pituitary-adrenal axis dysregulation, activate inflammatory responses, and contribute functional impairment^22–25^. Depressed and anxious patients have poor prognosis, with higher severity, greater chronicity, and longer treatment time. Also, depression is a known risk factor for noncompliance with medical treatment, and can be a serious concern in T2DM patients who require a higher degree of self-management. Cognitive deficits have been identified in 43% of T2DM individuals^26^, and deficits included in various domains, including psychomotor speed, executive function, verbal and working memory, processing speed, complex motor functioning, immediate and delayed recall, verbal fluency, visual retention, and attention^27–30^. In addition to depression and anxiety, impaired cognition has a negative impact on day-to-day self-care abilities and activities^31^. The presence of cognitive decline and depression and anxiety in T2DM patients worsens prognosis of underlying disease, impacts compliance to medical treatment^32^, decreases the quality of life, and increases morbidity and mortality. Although gray matter volumes are shown to be reduced in specific areas and its association with cognition has been observed in previous studies^7,10,33,34^, the relationships between brain structural status with depression and anxiety symptoms in T2DM are still unclear.

In the present study, our aim was to examine regional brain gray matter volume in T2DM patients compared to healthy control subjects, and assess its associations with cognition, depression, and anxiety symptoms, in T2DM subjects. Since cognition, anxiety, and depressive symptoms are common in T2DM, we hypothesized that regional brain gray matter volumes will be reduced in brain sites that are involved in cognition, depression, and anxiety regulation in T2DM compared to control subjects, and significant associations will emerge between symptom scores and gray matter volumes in those regulatory sites in T2DM patients.

## Results

### Demographics, microvascular complication, mood, and cognitive variables

There were no significant differences in age (p  =  0.08), sex (p  =  0.42), handedness (p = 0.71), and socioeconomic status (p = 0.97) appeared between T2DM patients and healthy controls (Table 1). Ethinicity varied between T2DM patient and control subjects (p = 0.004) (Table 1). Body-mass-index (p = 0.001) and systolic blood pressure (p = 0.02) values were significantly higher, and education levels were lower in T2DM patients over control subjects (p = 0.02) (Table 1). Nine T2DM patients had retinopathy, among whom five had diabetes opthalmologic disease only, three patients had other retinal disorders only, and one patient had diabetes opthalmologic disease, retinal disorders, and retinal detachment. Five T2DM patients had nephropathy, among whom one subject had diabetic nephropathy only, two patients had diabetic nephropathy and urine protein levels greater than 30 mg/g of creatinine, one patient had diabetic nephropathy, and serun creatinine level greater than 2.0 mg/dL, one patient had diabetic nephropathy, urine protein levels greater than 30 mg/g of creatinine, chronic renal failure, and serun creatinine level greater than 2.0 mg/dL. Three T2DM patients had diabetic neuropathy, and one patient had peripheral vascular disease. The HDL cholesterol levels of T2DM patients were 56.8 ± 19.9 mg/dL (n = 25). T2DM patients had significantly higher depression [increased BDI-II] (p  =  0.001) and greater anxiety [increased BAI] (p = 0.003) scores over healthy control subjects (Table 1). In addition, global cognition scores [decreased MoCA] were significantly reduced in T2DM patients compared to control subjects (p  =  0.002), with significant differences observed in language sub-domain (p  <  0.001; Table 1). Details of other variables are outlined in Table 1.

**Table 1:** Demographics and other variables of T2DM and control subjects.

Variables	T2DM n = 34 (Mean ± SD)	Controls n = 88 (Mean ± SD)	P values
Age (years)	56.8 ± 7.1	54.4 ± 5.1	0.08
Sex [male] (%)	15 (44%)	46 (52%)	0.42
BMI	29.8 ± 5.7	26.1 ± 3.5	0.001
Handedness [L/R/ambidex]	(n = 27) [2/25/0]	(n = 81) [6/73/2]	0.71
Edu level (years)	15.4 ± 2.1	16.6 ± 2.7	0.02
Ethnicity	White, 8 (24%); Hispanic, 12 (35%); Asian, 9 (26%) and Others, 5 (15%)	White, 37 (42%); Hispanic, 12 (14%); African American, 12 (14%); Asian, 22 (25%) and Others, 5 (5%)	0.004
Socioeco (Annual household income)	$ 94701.9 ± 35197.5 (n = 29)	$ 95072.9 ± 41155.2 (n = 85)	0.97
Systolic BP	126.0 ± 12.2	118.8 ± 16.7	0.02
Diastolic BP	75.2 ± 9.0	77.1 ± 15.0	0.50
Dur of T2DM	10.5 ± 7.7	—	—
HbA1c	7.4 ± 1.4% (57 ± 15.3 mmol/mol)	—	—
BAI	6.6 ± 7.6	2.5 ± 3.3	0.003
BDI-II	7.7 ± 6.7 (n = 32)	3.0 ± 4.0	0.001
Total MoCA	25.4 ± 2.3	26.9 ± 2.4	0.002
MoCA: Visuo	4.1 ± 1.0	4.5 ± 0.9	0.07
MoCA: Naming	2.9 ± 0.3	2.9 ± 0.3	0.55
MoCA: Attention	5.1 ± 1.0	5.4 ± 0.8	0.15
MoCA: Language	1.8 ± 1.1	2.6 ± 0.7	<0.001
MoCA: Abstract	1.9 ± 0.4	2.0 ± 0.1	0.14
MoCA: Del Recall	3.4 ± 1.2	3.5 ± 1.4	0.76
MoCA: Orient	6.0 ± 0.2	6.0 ± 0.1	0.83

SD = standard deviation; BMI = body mass index; Edu = education; Socioeco = socioeconomic status; BP = blood pressure; Dur = duration; BDI-II = Beck depression inventory II; BAI = Beck anxiety inventory; MoCA= Montreal cognitive assessment; Visuo = visuospatial; Abstract = abstraction; Del = delayed; Orient = orientation.

### Regional gray matter volume loss in T2DM

**Figure 1:**
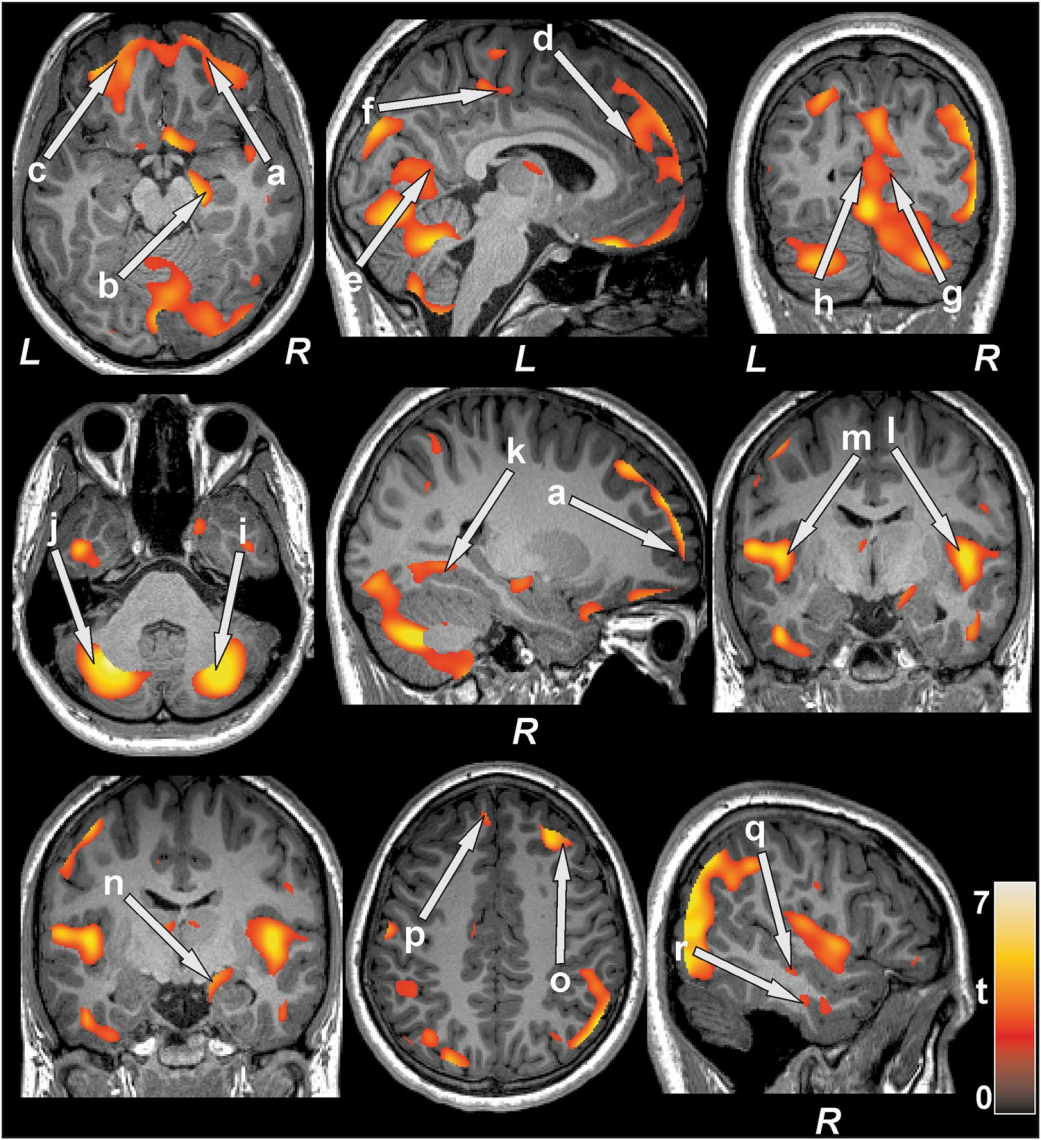
Brain regions with reduced gray matter volume in T2DM patients over controls after controlling for age and sex (FRD corrected, p < 0.01). These sites with reduced gray matter volume included the bilateral prefrontal cortices (a,c), right hippocampus (b), left anterior (d), mid (f) and posterior (e) cingulate, bilateral lingual gyrus (g,h), bilateral cerebellar cortices (i,j), right parahippocampal gyrus (k), bilateral anterior insula (l,m), right amygdala (n), right mid (o) and left superior (p) frontal cortices, right inferior (r) and mid (q) temporal gyrus. All images are in neurological convention (L  =  left; R  =  right). Color bar indicates *t*-statistic values.

Multiple brain areas showed reduced regional gray matter volume in T2DM compared to control subjects (Fig. 1; covariates, age and sex), including the bilateral prefrontal cortices (a, c), para-hippocampal gyrus (k), cerebellar cortex (i, j), vermis, brainstem, and bilateral cerebellar tonsil. The decreased gray matter volume was remarkable for the bilateral anterior (l, m) and posterior insular cortices, anterior (d), mid (f), and posterior (e) cingulate gyri, hippocampus (b), amygdala (n), caudate, basal-forebrain, thalamus, putamen, lingual gyrus (g, h), bilateral pre- and post-central gyrus, inferior, mid (o) and superior frontal cortex (p), inferior, mid, and superior occipital, superior parietal, and inferior (r), mid (q), and superior temporal gyrus. None of the brain regions showed increased gray matter volume in T2DM compared to control subjects. The regional brain gray matter volumes of T2DM and control subjects and effect sizes are tabulated in Table 2.

**Table 2:** Regional brain gray matter volume values (mean ± SD, mm^3^) of T2DM patients and control subjects and effect sizes.

Brain regions	T2DM (n = 34)	Control (n = 88)	P- values	Voxel Count	Effect Sizes
Left Prefrontal Cortex	0.35 ± 0.05	0.38 ± 0.05	<0.001	159	0.60
Right Prefrontal Cortex	0.37 ± 0.05	0.41 ± 0.05	<0.001	329	0.80
Brain Stem	0.31 ± 0.05	0.35 ± 0.05	<0.001	72	0.80
Right Basal Forebrain	0.35 ± 0.03	0.38 ± 0.03	<0.001	113	1.00
Left Caudate	0.34 ± 0.04	0.37 ± 0.04	0.001	78	0.75
Right Caudate	0.35 ± 0.04	0.39 ± 0.04	<0.001	869	1.00
Left Cerebellar Tonsil	0.39 ± 0.06	0.44 ± 0.07	<0.001	450	0.77
Right Cerebellar Tonsil	0.39 ± 0.07	0.44 ± 0.07	0.001	272	0.71
Left Cerebellar Cortex	0.50 ± 0.05	0.55 ± 0.05	<0.001	19817	1.00
Right Cerebellar Cortex	0.51 ± 0.05	0.56 ± 0.05	<0.001	16370	1.00
Cerebellar Vermis	0.43 ± 0.05	0.46 ± 0.05	<0.001	991	0.60
Right Hippocampus	0.40 ± 0.04	0.44 ± 0.04	<0.001	684	1.00
Right Amygdala	0.40 ± 0.04	0.43 ± 0.04	<0.001	132	0.75
Left Anterior Insula	0.46 ± 0.05	0.50 ± 0.05	<0.001	359	0.80
Right Anterior Insula	0.45 ± 0.05	0.49 ± 0.05	<0.001	611	0.80
Left Posterior Insula	0.45 ± 0.05	0.49 ± 0.05	<0.001	976	0.80
Right Posterior Insula	0.42 ± 0.05	0.46 ± 0.05	<0.001	2103	0.80
Left Lingual Gyrus	0.42 ± 0.05	0.46 ± 0.05	<0.001	3704	0.80
Right Lingual Gyrus	0.46 ± 0.04	0.50 ± 0.04	<0.001	2947	1.00
Left Anterior Cingulate	0.39 ± 0.05	0.43 ± 0.05	0.001	116	0.80
Right Anterior Cingulate	0.39 ± 0.05	0.42 ± 0.05	0.001	73	0.60
Right Posterior Cingulate	0.39 ± 0.04	0.42 ± 0.04	<0.001	61	0.75
Left Parahipp Gyrus	0.37 ± 0.05	0.41 ± 0.05	<0.001	89	0.80
Right Parahipp Gyrus	0.36 ± 0.03	0.38 ± 0.03	<0.001	320	0.67
Left Thalamus	0.32 ± 0.05	0.36 ± 0.05	<0.001	547	0.80
Right Thalamus	0.34 ± 0.05	0.38 ± 0.05	<0.001	935	0.80
Left Precentral Gyrus	0.31 ± 0.04	0.35 ± 0.04	<0.001	2054	1.00
Right Precentral Gyrus	0.32 ± 0.04	0.36 ± 0.04	<0.001	198	1.00
Left Postcentral Gyrus	0.31 ± 0.04	0.35 ± 0.04	<0.001	1040	1.00
Right Postcentral Gyrus	0.32 ± 0.04	0.35 ± 0.04	<0.001	399	0.75
Left Inf Frontal Gyrus	0.35 ± 0.04	0.39 ± 0.04	<0.001	450	1.00
Left Inf Occ Gyrus	0.35 ± 0.05	0.40 ± 0.05	<0.001	323	1.00
Right Inf Occ Gyrus	0.36 ± 0.04	0.40 ± 0.04	<0.001	3695	1.00
Left Inf Temp Gyrus	0.45 ± 0.05	0.49 ± 0.05	<0.001	1087	0.80
Right Inf Temp Gyrus	0.43 ± 0.05	0.48 ± 0.05	<0.001	1303	1.00
Left Mid Frontal Cortex	0.33 ± 0.04	0.36 ± 0.04	<0.001	68	0.75
Right Mid Frontal Cortex	0.33 ± 0.04	0.37 ± 0.04	<0.001	1959	1.00
Left Mid Occ Gyrus	0.34 ± 0.04	0.38 ± 0.04	<0.001	781	1.00
Right Mid Occ Gyrus	0.36 ± 0.04	0.40 ± 0.04	<0.001	3597	1.00
Left Mid Temp Gyrus	0.36 ± 0.04	0.40 ± 0.04	<0.001	444	1.00
Right Mid Temp Gyrus	0.37 ± 0.04	0.41 ± 0.04	<0.001	2199	1.00
Left Sup Frontal Cortex	0.31 ± 0.04	0.34 ± 0.04	<0.001	2346	0.75
Right Sup Frontal Cortex	0.31 ± 0.04	0.35 ± 0.04	<0.001	410	1.00
Left Sup Occ Cortex	0.31 ± 0.04	0.34 ± 0.04	<0.001	1170	0.75
Right Sup Occ Cortex	0.34 ± 0.05	0.37 ± 0.05	<0.001	245	0.60
Left Sup Parietal Cortex	0.32 ± 0.04	0.36 ± 0.04	<0.001	1859	1.00
Right Sup Parietal Cortex	0.34 ± 0.05	0.38 ± 0.05	<0.001	1136	0.80
Left Sup Temp Cortex	0.42 ± 0.06	0.47 ± 0.06	<0.001	624	0.83
Right Sup Temp Cortex	0.41 ± 0.06	0.45 ± 0.06	<0.001	483	0.67

SD = Standard deviation; T2DM = Type 2 diabetes mellitus; Mid = Middle; Parahipp = Para-hippocampal; Inf = Inferior; Occ = Occipital; Temp = Temporal; Sup = Superior.

### Correlations between gray matter volumes and BAI, BDI-II, MoCA scores in T2DM

**Figure 2:**
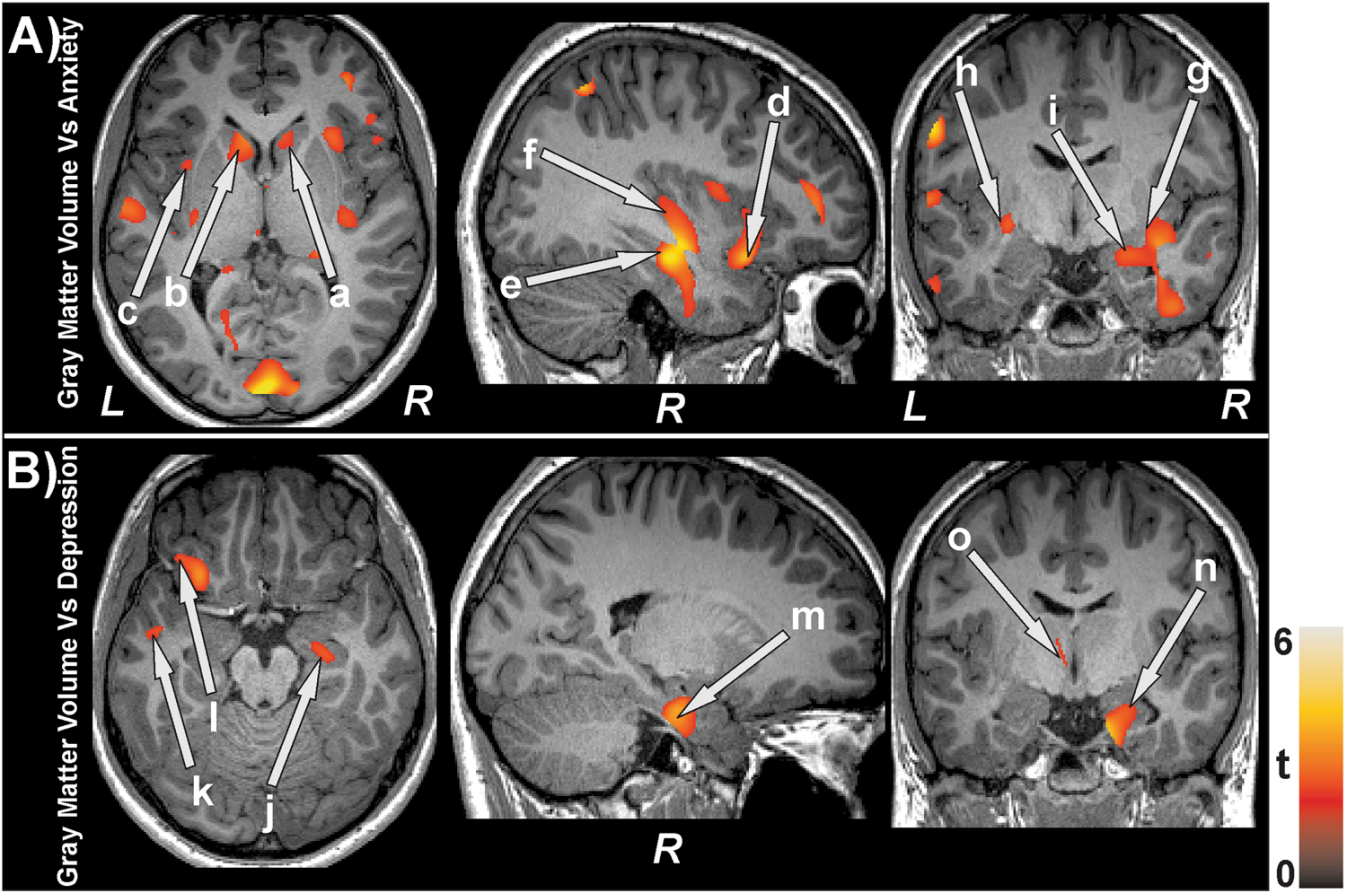
Negative correlations emerged between gray matter volume and behavioral symptoms in T2DM subjects. Negative correlations appeared between gray matter volume and anxiety scores at the bilateral caudate (a,b), anterior (c,d), and posterior (f) insula, and hippocampus (e), bilateral putamen (g,h), amygdala (i) and with depression levels at right hippocampus (j), left superior temporal (k) and prefrontal (l) cortices, right parahippocampus (m), right amygdala (n), and left thalamus (o). Figure conventions are same as in Fig. 1.

**Figure 3:**
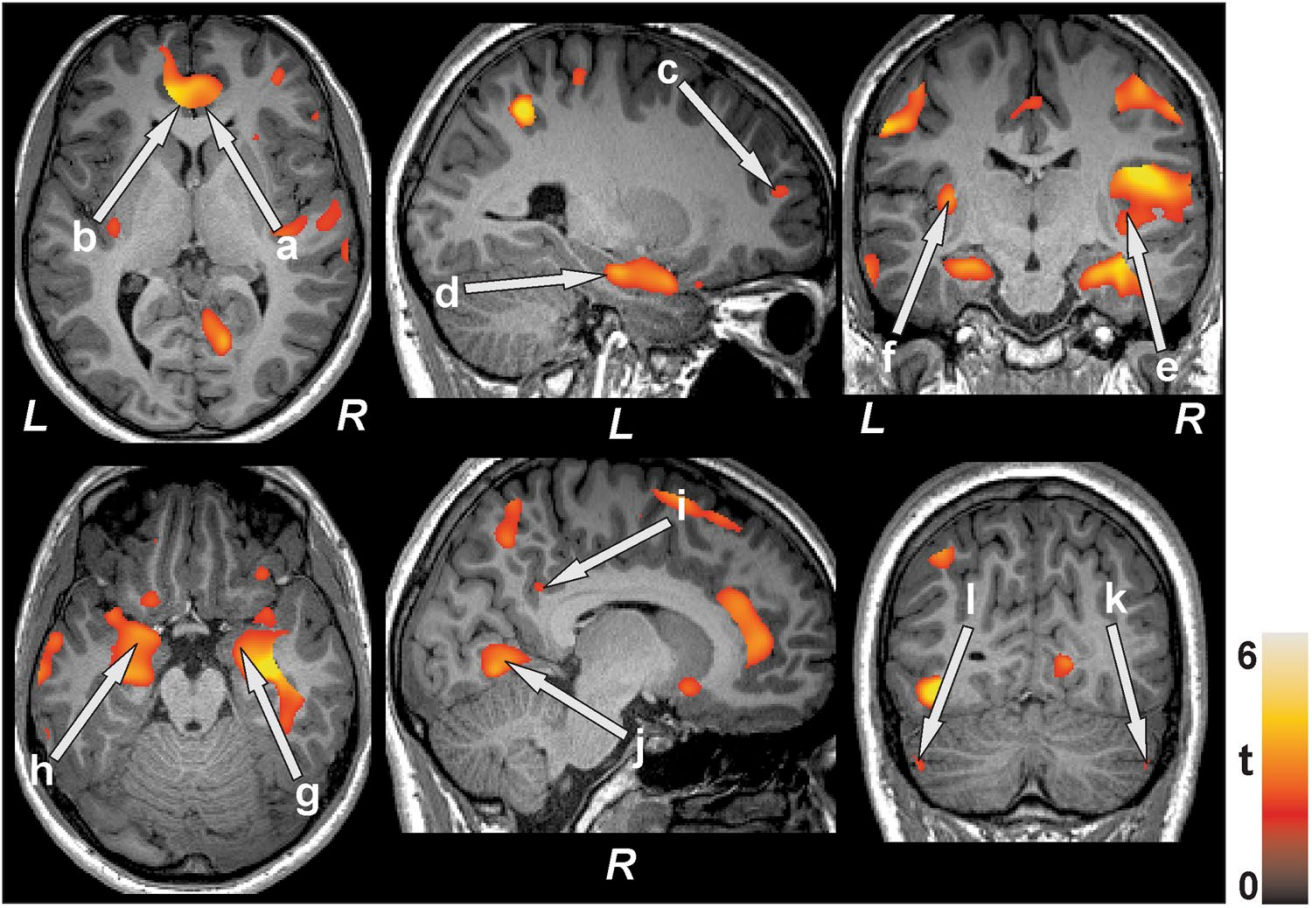
Cognition showed positive associations with gray matter volume in T2DM subjects in several brain sites. Positive correlations appeared between gray matter volume and MoCA scores at the bilateral anterior (a,b) and right posterior (i) cingulate, prefrontal cortices (c), hippocampus (d), bilateral posterior insula (e,f), bilateral amygdala (g,h), and right lingual gyrus (j) and bilateral cerebellar cortices (k,l). Figure conventions are same as in Fig. 1.

Anxiety scores showed negative associations with gray matter volumes (Fig. 2A) at the bilateral caudate (a, b), bilateral mid, left superior and right inferior frontal cortices, anterior, mid, posterior cingulate, putamen (g, h), hippocampus (e) and parahippocampus, anterior (c, d) and posterior insula (f), amygdala (i), pallidum, thalamus, basal forebrain, lingual gyrus, post and precentral cortices, inferior, mid, and superior occipital cortices, inferior, mid, and superior temporal cortices, superior parietal cortices, and prefrontal cortices in T2DM subjects. Negative relationships were observed in T2DM subjects between depression scores and gray matter volumes (Fig. 2B) at the amygdala (n), inferior frontal cortices, thalamus (o), anterior insula, hippocampus (j), para-hippocampal gyrus (m), prefrontal cortices (l), inferior, mid, and superior occipital cortices, and inferior, mid, superior (k) temporal cortices. Global MoCA values (cognition scores) showed positive correlations with gray matter volumes (Fig. 3) at the bilateral cerebellar cortices (k, l) and vermis, basal forebrain, anterior, and posterior (e, f) insula, pre- and post-central gyrus, middle and superior frontal cortices and prefrontal cortices (c), bilateral para-hippocampal gyrus, anterior (a, b), mid, and posterior (i) cingulate, hippocampus (d), amygdala (g, h), putamen, superior parietal gyrus, inferior, and mid occipital and inferior, mid, and superior temporal gyrus, and lingual gyrus (j). The correlation coefficients of gray matter volumes for all brain regions showing significant association with anxiety, depression, and cognitive scores are tabulated in Table 3.

**Table 3:** Correlation between regional gray matter volume and mood and cognition in T2DM subjects.

Measures	Brain Regions	Correlation Coefficient	P- values	Voxel Count
Anxiety (BAI, n = 34)	Left Caudate	−0.504	0.003	1421
	Right Caudate	−0.470	0.007	294
	Left Hippocampus	−0.545	0.001	802
	Right Hippocampus	−0.582	<0.001	1723
	Left Putamen	−0.530	0.002	578
	Right Putamen	−0.499	0.004	110
	Left Thalamus	−0.485	0.005	350
	Right Thalamus	−0.488	0.005	80
	Left Basal Forebrain	−0.532	0.002	249
	Right Anterior Cingulate	−0.477	0.006	156
	Right Mid Cingulate	−0.485	0.005	116
	Left Posterior Cingulate	−0.494	0.004	368
	Left Anterior Insula	−0.525	0.002	788
	Right Anterior Insula	−0.556	0.001	2729
	Left Posterior Insula	−0.508	0.003	224
	Right Posterior Insula	−0.509	0.003	703
	Left Lingual Gyrus	−0.542	0.001	539
	Right Lingual Gyrus	−0.566	0.001	1205
	Left Parahippocampus	−0.510	0.003	148
	Right Amygdala	−0.485	0.005	270
	Left Inferior Temporal	−0.530	0.002	340
	Right Inferior Temporal	−0.532	0.002	717
	Left Mid Temporal	−0.503	0.003	814
	Right Mid Temporal	−0.533	0.002	639
	Left Superior Temporal	−0.504	0.003	208
	Right Superior Temporal	−0.503	0.003	366
	Left Prefrontal	−0.503	0.003	76
	Right Ventral Medial Prefrontal	−0.485	0.005	96
	Right Inferior Frontal	−0.522	0.002	125
	Left Mid Frontal	−0.518	0.002	157
	Right Mid Frontal	−0.552	0.001	232
	Left Superior Frontal	−0.527	0.002	725
	Left Mid Occipital	−0.493	0.004	87
	Right Mid Occipital	−0.583	<0.001	414
	Left Superior Occipital	−0.588	<0.001	840
	Right Superior Occipital	−0.563	0.001	801
	Left Superior Parietal	−0.555	0.001	560
	Right Superior Parietal	−0.634	<0.001	1343
	Left Precentral Gyrus	−0.495	0.004	82
	Left Postcentral Gyrus	−0.558	0.001	505
Depression (BDI-II, n = 32)	Left Prefrontal	−0.548	0.002	85
	Right Hippocampus	−0.533	0.002	947
	Left Thalamus	−0.483	0.007	114
	Right Parahippocampus	−0.558	0.001	794
	Right Inferior Frontal	−0.507	0.004	70
	Left Mid Temporal	−0.494	0.006	48
	Right Mid Temporal	−0.477	0.008	83
	Left Superior Temporal	−0.553	0.002	286
	Right Inferior Occipital	−0.514	0.004	93
	Right Mid Occipital	−0.529	0.003	151
	Left Superior Occipital	−0.489	0.006	63
Cognition (MoCA, n = 34)	Left Amygdala	0.517	0.002	849
	Right Amygdala	0.535	0.002	861
	Left Anterior Cingulate	0.586	<0.001	3407
	Right Anterior Cingulate	0.547	0.001	3371
	Left Posterior Cingulate	0.495	0.004	817
	Left Mid Cingulate	0.499	0.004	298
	Left Hippocampus	0.543	0.001	1450
	Right Hippocampus	0.598	<0.001	1623
	Left Anterior Insula	0.486	0.005	136
	Left Posterior Insula	0.519	0.002	609
	Right Posterior Insula	0.545	0.001	978
	Prefrontal Cortices	0.559	0.001	1518
	Right Putamen	0.482	0.005	103
	Right Lingual Gyrus	0.557	0.001	1068
	Left Parahippocampus	0.484	0.005	325
	Right Parahippocampus	0.491	0.004	499
	Left Cerebellar Cortices	0.482	0.005	153
	Right Cerebellar Cortices	0.465	0.007	83
	Left Mid Frontal	0.506	0.003	846
	Right Mid Frontal	0.586	<0.001	638
	Left Superior Frontal	0.548	0.001	2585
	Right Superior Frontal	0.550	0.001	1630
	Left Superior Parietal	0.594	<0.001	1177
	Right Superior Parietal	0.595	<0.001	1507
	Right Inferior Temporal	0.607	<0.001	1186
	Left Mid Temporal	0.565	0.001	2397
	Right Mid Temporal	0.483	0.005	119
	Right Superior Temporal	0.504	0.003	876
	Left Inferior Occipital	0.559	0.001	672
	Left Mid Occipital	0.521	0.002	155
	Right Precentral Gyrus	0.534	0.002	1129
	Left Postcentral Gyrus	0.618	<0.001	2219
	Right Postcentral Gyrus	0.590	<0.001	1147

BAI = Beck Anxiety Inventory; BDI-II = Beck Depression Inventory II; MoCA = Montreal Cognitive Assessment.

### Overlap between ANCOVA (gray matter volumes; T2DM vs control) and partial correlation analyses (gray matter volumes vs BAI, BDI-II, and MoCA scores)

**Figure 4:**
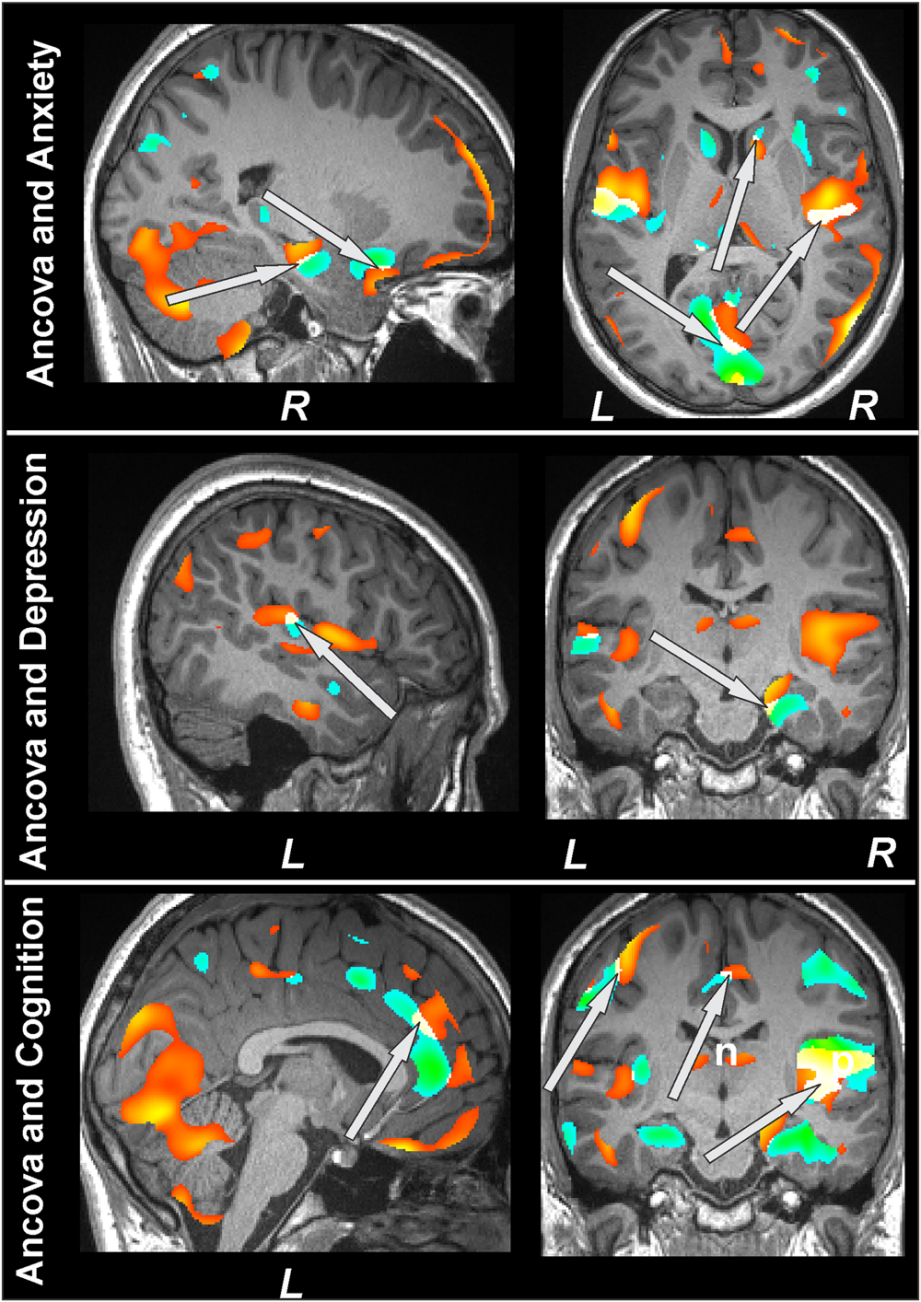
Significant clusters from ANCOVA (gray matter volumes; T2DM vs controls) and correlational analyses (gray matter volumes vs BAI, BDI-II, and MoCA scores; T2DM). Multiple sites that are involved in cognition (hippocampus, prefrontal cortex, parahippocampus, cingulate, insula, and cerebellum), anxiety (hippocampus, cingulate, insula, and amygdala), and depression (hippocampus, cingulate, and insula) regulation overlapped between group differences and correlation analyses that are marked with arrows.

The overlap between ANCOVA findings for gray matter volume between T2DM patients and control subjects and partial correlations between gray matter volumes and mood and cognitive symptoms in multiple specific brain regions that regulate cognition (hippocampus, prefrontal cortex, and cerebellum), anxiety (hippocampus, cingulate, insula, and amygdala), and depressive symptoms (hippocampus, cingulate, and insula) are shown in Fig. 4.

## Discussion

Prior studies have been based on either gray matter changes, or gray matter and cognitive changes, or gray matter and vaso-reactivity, or global gray matter changes associated with depression, anxiety, and cognition in T2DM individuals. In this study, we demonstrated significant regional gray matter volume differences in depression (hippocampus, cingulate, insula, thalamus, and parahippocampus), anxiety (hippocampus, cingulate, insula, and amygdala), and cognitive (hippocampus, prefrontal cortex, and cerebellum) control areas, and associations between gray matter volume changes and cognitive, depression, and anxiety symptom scores in T2DM subjects. The performance of T2DM patients on MoCA, was significantly lower compared to control subjects, and BDI-II and BAI in T2DM patients showed higher scores indicating those symptoms. Within the T2DM group, cognitive function was positively related to the gray matter volume, and negative associations were found between gray matter volume and depression and anxiety scores. Brain sites with gray matter volume loss differences between T2DM and controls overlapped with correlations between regional gray matter volumes and mood and cognitive symptom measures in T2DM patients. These data suggest that changes in cognition, depression, and anxiety are associated with tissue changes in those regulatory sites in the condition.

The pathophysiology underlying gray matter changes in T2DM subjects may involve an interplay between endocrinologic, metabolic, and vascular pathways^35^. T2DM is associated with cerebral energy homeostasis changes, which may induce inflammation and can substantially alter vascular physiology, including reduced endothelial dependent vasodilatation and deficits in cerebral vascular reactivity to CO_2_^36^. The altered cerebral vascular reactivity, which is key for maintaining an optimal environment for neuronal survival, may contribute to regional gray matter changes. In addition, small vessel occlusion may also lead to observed regional brain changes in T2DM subjects. Chronic hyperglycemia, as observed in T2DM subjects, increases the formation of advanced glycation end products (AGEs) through non-enzymatic glycation, and the interaction of AGEs with the receptors of AGEs elicits production of reactive oxygen species, which promote oxidative stress, leading to inflammation along with other processes^37^. Mitochondrial oxidative stress alters the endoplasmic reticulum signaling, that lead to activation of major cell-damaging pathways and consequent neuronal cell damage, resulting to regional gray matter volume changes as observed here. Insulin and insulin receptors are also abundant in the brain, and play a major role in modulating cerebral glucose metabolism. These receptors are selectively distributed across the brain, with high concentrations in the cerebral cortex, hippocampus, and amygdala^38^; decline in insulin transport across the blood brain barrier and insulin resistance in areas with high receptor concentration may impair regional glucose metabolism and may contribute to gray matter volume changes. Our findings include significant gray matter changes in temporal, frontal, and occipital lobes, sites that have shown associations with diabetic metabolic disturbance, cerebral vasoreactivity^39^, and decreased functional magnetic resonance imaging based amplitude of low frequency fluctuation values in T2DM patients^40^.

Mood and anxiety changes are characterized by a variety of neuroendocrine, neurotransmitter, and neuroanatomical abnormalities. A significant degree of interconnectivity between neurotransmitters and neuropeptides exist containing circuits in limbic, brainstem, and higher cortical areas. Multiple sites, including the hippocampus, amygdala, insula, and anterior cingulate cortices showed tissue changes in T2DM subjects. A primary alteration in brain neurotransmitter signaling may result from the underlying T2DM condition and may affect the mood and anxiety status. Hyperglycemia interferes with the metabolism of monoamine neurotransmitters, including serotonin, norepinephrine, and various neuropeptides (substance P, somatostatin, neuropeptide Y, met-enkephalin, vasoactive intestinal peptide, beta-endorphin, and vasopressin), resulting in a multitude of effects on behavior, mood, appetite, and pain perception.

The prefrontal cortex (PFC) is responsible for predicting consequences for potential behaviors and understanding and moderating social behavior, and ventral medial region of PFC codes information, controls impulses, and regulates mood behavior via inhibitory top-down control of emotional-processing structures^41^. Gray matter volume at these regions had negative correlations with depression, as well as anxiety scores in T2DM subjects in this study. The limbic system responsible for emotional-processing includes the insular cortex, dysfunction of this structure can affect the saliency network and might contribute to the high anxiety symptoms^42^, and insular gray matter volume showed negative associations with anxiety scores in our study. The hippocampus is another limbic structure, the volume and neurogenesis of this region have been implicated in stress sensitivity and resiliency in relationship to mood and anxiety deficits, and negative associations were observed between hippocampal gray matter volume and anxiety and depression scores. The hippocampus has tonic inhibitory control over the hypothalamic stress-response system and plays a negative feedback role for the hypothalamic-pituitary-adrenal axis. The hypothalamus and thalamus are highly interconnected, and gray matter volume at thalamus showed negative correlations with depression and anxiety index here. In addition, the hippocampus/parahippocampal gyrus is a key structure in the limbic-cortical dysregulation model in major depression^43,44^, and gray matter volume at para-hippocampal gyrus was negatively associated with depressive and anxiety symptoms. The fronto-striatal pathway that connects the frontal lobe with the striatum and mediates behavioral functions and the gray matter volume at frontal lobe and striatum showed negative associations with depression and anxiety scores in this study. Dorsolateral PFC-posterior cingulate connectivity is associated with anxiety^45^ and gray matter volume was negatively associated with anxiety scores in our study.

Altered functional activity has been observed in dorsolateral PFC, anterior cingulate, and insula during emotional interference that has been associated with worry^46^. The anticipation of negative outcomes recruits a neural network that includes the anterior cingulate, insula, amygdala, dorsolateral PFC, and parahippocampal gyrus^47,48^. The insula play a significant role in the effective and interoceptive processing^49^, along with dysfunctional anticipatory processing of anxiety^50^. Insula together with anterior cingulate constitute a fear network, and cingulate is also involved in conflict-monitoring and fear learning^51^. The prefrontal cortex is known to be activated upon the presentation of emotional distractors during a working memory task^52^. All these regions showed significant association between gray matter volume and anxiety scores in our T2DM patients suggesting higher anxiety levels with decreased gray volumes in patients.

T2DM is associated with accelerated cognitive decline^53^, and an increased risk of dementia^54^. However, the exact pathophysiology of cognitive dysfunction in T2DM is not completely understood, but may include hyperglycemia, vascular disease, and insulin resistance, as indicated above. Inherent connections of the frontal lobe form vital feed-forward and feed-backward circuits from the prefrontal information processing center. The PFC is an interconnected set of neocortical areas that have connectivity with higher-order regions and are implicated in executive functioning, memory, intelligence, language, and visual search; gray matter volume in frontal and prefrontal cortices showed positive associations with MoCA scores in this study. A dynamic relationship between PFC and anterior cingulate exist, with the primary role of anterior cingulate being conflict resolution and providing input to the top-down attentional dorsolateral system that plays a vital role in cognitive control. Gray matter volume in the anterior cingulate showed significant positive associations with cognitive scores in our data that may support the above notion. The ventral posterior cingulate cortex is highly integrated with the default mode network, and is involved in internally-directed cognition, such as memory retrieval and planning, and the dorsal posterior cingulate shows a highly complex pattern of connectivity, with prominent connections to the frontal lobe, and is involved in controlling attentional focus^55^; the posterior cingulate cortices showed associations with cognition scores in this study. In addition, the PFC and hippocampus are functionally-interconnected^56^, connections from the prefrontal cortex to the entorhinal, perirhinal, and parahippocampal cortices and to the hippocampus and the reciprocal connections from the hippocampus back to the prefrontal cortex, indicating a major role of the hippocampus in cognition^57^, including the memory consolidation, spatial cognition, temporal information processing, and sequencing. The gray matter volume in the hippocampus and para-hippocampus showed positive associations with cognition here. The lingual gyrus is an early visual processing area associated with letter processing^58^, visual imagery^59^, and affects a higher ability in divergent thinking tasks requiring visual imagination, and gray matter volume in this region correlated with cognition in this study. Cerebellum contributes to cognitive processing, in addition to motor coordination roles^60^. Abnormalities of the posterior cerebellum has been associated with cognitive difficulties in several domains, including executive, visuospatial, language, and memory dysfunctions^61–63^. Cerebellar activation has been observed during multiple cognitive tasks, including language tasks in functional imaging experiments^60^. Also, the cerebellum is extensively interconnected with the cerebral hemisphere both in feed-forward and feed-backward directions, and provides a structural basis for functional roles of the cerebellum in cognitive functions, as indicated here with gray matter volume and cognitive score associations.

There are a few limitations in this study. This study was limited to a small sample size of T2DM subjects that may affect the statistical analyses and comprehensive interpretation of findings, although reasonable number of control subjects were included that provided sufficient statistical power. We used MoCA, BDI-II, and BAI screening instruments to identify cognitive impairment and symptoms of depression and anxiety, elaborated clinical tests should be used for future studies. Blood glucose levels were examined for some control subjects before MRI; however, most of the control subjects self-confirmed that they did not have T2DM and can be considered as a limitation. The occurrence of hypoglycemic events up to 24 h prior to testing can affect cognitive abilities of T2DM patients, but such hypoglycemic event data were lacking. Also, correlation findings were not FDR corrected for multiple comparisons due to small sample sizes (34 T2DM patients) and can be considered as a limitation. Also, this is a cross-sectional study, therefore causality cannot be established. A longitudinal research with a larger sample size is needed to evaluate the findings from this present study in consideration of important clinical variables such as HbA1c in T2DM, and to determine whether these brain abnormalities are reversible with improvements in glycemic control.

To conclude, T2DM patients showed significant brain structural changes in widespread areas, including the prefrontal cortices, hippocampus, parahippocampus, amygdala, insula, cingulate, caudate, thalamus, and cerebellum, sites that are involved in depression, anxiety, and cognition regulation. In addition, T2DM patients exhibited significant anxiety and depression symptoms and impairment in cognitive abilities, and brain regions regulating these functions showed significant gray matter volume loss. The findings indicate that cognitive deficits and mood disorders in T2DM are associated with brain structural damage.

## Materials and Methods

### Subjects

We studied 34 T2DM and 88 healthy non-diabetic control subjects. Demographic, physiologic, neuropsychologic, and cognitive data are summarized in Table 1. All T2DM subjects were recruited from the Gonda Diabetes Center at the University of California Los Angeles (UCLA) and the surrounding community. All T2DM subjects were on diabetes medication (e.g., metformin, canagliflozin, exenatide, repaglinide, glyburide, sitagliptin, glipizide); twelve T2DM subjects were on insulin and twenty-one were on high blood pressure and/or cholesterol medications. Inclusion criteria for the T2DM subjects were to be clinically diagnosed with the condition, on stable T2DM medication (no changes in medications or dosages in previous 6 weeks), and able to lay flat. Control subjects were healthy and recruited via flyers from the UCLA campus and the West Los Angeles area. Some control subjects were assessed for A1C levels using finger stick blood method of point-of-care HbA1C testing with the A1cNow instrument, and majority of control subjects self-confirmed that they did not have T2DM. Control subjects were not on anti-hypertensive medication therapy, and without any known neurological or psychiatric conditions, cardiovascular, or sleep disorder issues that would introduce brain injury or drug dependency, e.g., tobacco or cocaine use that would modify autonomic control and brain tissue. Study exclusion criteria included a history of stroke, heart failure, diagnosed brain condition, metallic implants, claustrophobia, or body weight more than 160 kg (scanner limitation). The diabetes complications severity index (DCSI) data were collected from T2DM patients’ clinical charts to assessmicrovascular complication status. The HDL cholesterol levels were also noted from patients’ clinical chart. All subjects provided informed written consent prior to the study; the protocol was approved by the UCLA Institutional Review Board. All methods were performed in accordance with the relevant guidelines and regulations.

### Assessment of mood and anxiety

Depressive and anxiety symptoms were evaluated using the Beck Depression Inventory (BDI-II)^64^ and Beck Anxiety Inventory (BAI)^65^, respectively. These questionnaires are self-administered, with 21 questions in each inventory, and scores for each question varying from 0–3, with each total score ranging from 0–63 depending on severity of symptoms. T2DM or control subjects with values >9 for BDI-II or BAI were considered to have depressive or anxiety symptoms, respectively.

### Cognitive assessment

The Montreal Cognitive assessment (MoCA) test was used for rapid evaluation of various cognitive domains in all subjects, including attention and concentration, executive functions, memory, language, visuo-constructional skills, conceptual thinking, calculations, and orientation. A global MoCA score ≥26 was considered normal^66^.

### Socioeconomic status

Socioeconomic status was obtained from the American Community Survey data available on Population Studies Center, Institute for Social Research, based on residential postal codes of each subject and their annual household income was evaluated.

### Magnetic resonance imaging

Brain imaging data were collected using a 3.0-Tesla MRI scanner (Siemens, Magetom, Tim-Trio/Prisma, Erlangen, Germany). We collected two high-resolution T1-weighted image series using the magnetization-prepared rapid acquisition gradient-echo pulse sequence [repetition-time (TR) = 2200 ms; echo-time (TE) = 2.34/2.41 ms; inversion time = 900 ms; flip angle (FA) = 9°; matrix size = 320 × 320; field-of-view (FOV) = 230 × 230 mm^2^; slice thickness = 0.9 mm)]. Proton-density (PD) and T2-weighted images were collected using a dual-echo turbo spin-echo sequence in the axial plane (TR = 10,000 ms; TE1, 2 = 12, 123/124 ms; FA = 130°; matrix size = 256 × 256; FOV = 230 × 230 mm^2^; slice thickness = 3.5 mm). We visually assessed T1-, T2-, and PD-weighted images of all subjects for any major pathology, such as cystic lesions, infarcts, or tumors to subsequently exclude subjects if found with any abnormality. For any head-motion related or other imaging artifacts, we critically examined high-resolution T1-weighted images immediately after the scan, and repeated if necessary.

### Data processing and analyses

The statistical parametric mapping package (SPM12, http://www.fil.ion.ucl.ac.uk/spm/), MRIcroN, RESting-state fMRI data analysis Toolkit (REST) and MATLAB-based (The MathWorks Inc., Natick, MA, USA) custom software were used for data processing and analyses. Both high-resolution T1-weighted images were reoriented to remove any potential variation from head motion, and averaged to increase signal-to-noise ratio. The T1-weighted images were realigned in the space of the first series. Second T1-weighted image volume was realigned to first image volume using rigid-body transformation (FWHM, 3 mm) and averaged. The averaged T1-weighted images were partitioned into gray matter, white matter, and cerebrospinal fluid (CSF) tissue types. The Diffeomorphic Anatomical Registration Through Exponentiated Lie algebra algorithm (DARTEL) toolbox^67^ was used to generate the flow fields, which are nonlinear deformations applied for warping all the gray matter images to match each other and template images that were implemented for normalization of gray matter maps to Montreal Neurological Institute (MNI) space (voxel size: 1 × 1 × 1 mm^3^). The modulated and normalized maps were smoothed using a Gaussian filter, and the smoothed gray matter maps were used for further statistical analyses.

### Background image

The average T1-weighted images from one control subject was normalized to MNI space. The normalized images were used as background images for structural identification.

### Region-of-interest analyses

Region-of-interest (ROI) analyses were performed to calculate regional gray matter volumes to determine magnitude differences between groups, and correlation coefficients for each correlation analyses. Regional brain masks were created based on ROIs from Neuromorphometrics, Inc. (www.neuromorphometrics.com) and significant whole-brain voxel based gray matter volume differences between groups and significant correlation between gray matter volume of associated regions and behavioral scores. The ROI values were extracted using these regional masks of specific brain regions and smoothed gray matter volumetric maps of T2DM and controls.

### Statistical analyses

#### Demographics and other variables

The Statistical Package for the Social Sciences (IBM SPSS, v25.0, Armonk, NY, USA) was used for assessment of demographic, physiological, mood, and cognitive variables. Demographic and clinical variables were assessed by independent samples t-tests, and categorical variables were compared using the Chi-square test. A P-value of <0.05 was considered statistically significant.

### Regional brain gray matter volume changes between T2DM and controls

The smoothed whole-brain gray matter maps were compared between T2DM and control subjects using analysis of covariance [ANCOVA, SPM12; covariates, age and sex; false discovery rate (FDR) correction for multiple comparison, p < 0.01, minimum extended cluster size, 40 voxels). The FDR correction was performed using the REST toolbox based on the t-statistic maps obtained from SPM12. Brain clusters with significant differences between groups were overlaid onto background images for structural identification.

### Regional brain gray matter volumes and effect sizes

Regional gray matter volumes, calculated from ROI analyses, were examined for significant differences between T2DM and control subjects using ANCOVA (SPSS; covariates, age and sex) and calculate effect sizes. A p-value of <0.05 was chosen to establish statistical significance.

### Correlations between gray matter volumes and BAI, BDI-II, and MoCA scores in T2DM

Whole-brain gray matter maps were correlated voxel-by-voxel with BAI, BDI-II, and MoCA scores in T2DM subjects using partial correlations (SPM12; covariates, age and sex, uncorrected, p < 0.005, minimum extended cluster size, 40 voxels). Brain clusters showing significant correlations between gray matter volumes and BAI, BDI-II, and MoCA scores were overlaid on background images. To obtain region-specific correlation coefficient values, ROI values were obtained from specific brain regions that showed significant correlations in whole-brain voxel based correlation analyses, and examined with partial correlations (SPSS; covariates, age and sex, p < 0.05).

### Overlap between ANCOVA (Gray matter volumes; T2DM vs control) and partial correlation analyses (Gray matter volumes vs BAI, BDI-II, and MoCA scores)

Brain clusters with significant differences in gray matter volumes between T2DM patients and control subjects based on ANCOVA were overlaid onto background images. Similarly, brain clusters showing correlations between gray matter volumes and symptoms scores (BDI-II, BAI, and MoCA) in T2DM patients were overlaid onto background images. Common overlapping brain sites between group differences and correlation analyses were identified.

## References

[1] Tripathi BK, Srivastava AK (2006). Diabetes mellitus: complications and therapeutics. Med. Sci. Monit..

[2] Kaiser AB, Zhang N, Wouter VDP (2018). Global Prevalence of Type 2 Diabetes over the Next Ten Years (2018–2028). Diabetes.

[3] Kroner Z (2009). The relationship between Alzheimer’s disease and diabetes: Type 3 diabetes?. Altern. Med. Rev..

[4] Schmidt R (2004). Magnetic resonance imaging of the brain in diabetes: the Cardiovascular Determinants of Dementia (CASCADE) Study. Diabetes.

[5] den Heijer T (2003). Type 2 diabetes and atrophy of medial temporal lobe structures on brain MRI. Diabetologia.

[6] Chen Z, Li L, Sun J, Ma L (2012). Mapping the brain in type II diabetes: Voxel-based morphometry using DARTEL. Eur. J. Radiol..

[7] Moran C (2013). Brain atrophy in type 2 diabetes: regional distribution and influence on cognition. Diabetes Care.

[8] Zhang Y (2014). Gray matter volume abnormalities in type 2 diabetes mellitus with and without mild cognitive impairment. Neurosci. Lett..

[9] Wisse LE (2014). Global brain atrophy but not hippocampal atrophy is related to type 2 diabetes. J. Neurol. Sci..

[10] Espeland MA (2013). Influence of type 2 diabetes on brain volumes and changes in brain volumes: results from the Women’s Health Initiative Magnetic Resonance Imaging studies. Diabetes Care.

[11] Yau PL (2010). Preliminary evidence for brain complications in obese adolescents with type 2 diabetes mellitus. Diabetologia.

[12] Kumar R, Anstey KJ, Cherbuin N, Wen W, Sachdev PS (2008). Association of type 2 diabetes with depression, brain atrophy, and reduced fine motor speed in a 60- to 64-year-old community sample. Am. J. Geriatr. Psychiatry.

[13] Moulton CD, Costafreda SG, Horton P, Ismail K, Fu CH (2015). Meta-analyses of structural regional cerebral effects in type 1 and type 2 diabetes. Brain Imaging Behav..

[14] Alosco ML (2013). The adverse impact of type 2 diabetes on brain volume in heart failure. J. Clin. Exp. Neuropsychol..

[15] Brundel M (2010). Cerebral cortical thickness in patients with type 2 diabetes. J. Neurol. Sci..

[16] Kumar A (2008). Gray matter prefrontal changes in type 2 diabetes detected using MRI. J. Magn. Reson. Imaging.

[17] Novak V (2011). Adhesion molecules, altered vasoreactivity, and brain atrophy in type 2 diabetes. Diabetes Care.

[18] Gavard JA, Lustman PJ, Clouse RE (1993). Prevalence of depression in adults with diabetes. An epidemiological evaluation. Diabetes Care.

[19] Watari K (2006). Cognitive function in adults with type 2 diabetes and major depression. Arch. Clin. Neuropsychol..

[20] Roy T, Lloyd CE (2012). Epidemiology of depression and diabetes: a systematic review. J. Affect. Disord..

[21] Grigsby AB, Anderson RJ, Freedland KE, Clouse RE, Lustman PJ (2002). Prevalence of anxiety in adults with diabetes: a systematic review. J. Psychosom. Res..

[22] Varghese FP, Brown ES (2001). The Hypothalamic-Pituitary-Adrenal Axis in Major Depressive Disorder: A Brief Primer for Primary Care Physicians. Prim. Care Companion J. Clin. Psychiatry.

[23] Faravelli C (2012). Childhood stressful events, HPA axis and anxiety disorders. World J. Psychiatry.

[24] Salim S, Chugh G, Asghar M (2012). Inflammation in anxiety. Adv. Protein Chem. Struct. Biol..

[25] Felger JC (2019). Role of Inflammation in Depression and Treatment Implications. Handb. Exp. Pharmacol..

[26] Luchsinger JA (2007). Relation of diabetes to mild cognitive impairment. Arch. Neurol..

[27] Fontbonne A, Berr C, Ducimetiere P, Alperovitch A (2001). Changes in cognitive abilities over a 4-year period are unfavorably affected in elderly diabetic subjects: results of the Epidemiology of Vascular Aging Study. Diabetes Care.

[28] Grodstein F, Chen J, Wilson RS, Manson JE (2001). & Nurses’ Health, S. Type 2 diabetes and cognitive function in community-dwelling elderly women. Diabetes Care.

[29] Munshi M (2006). Cognitive dysfunction is associated with poor diabetes control in older adults. Diabetes Care.

[30] Reaven GM, Thompson LW, Nahum D, Haskins E (1990). Relationship between hyperglycemia and cognitive function in older NIDDM patients. Diabetes Care.

[31] Sinclair AJ, Girling AJ, Bayer AJ (2000). Cognitive dysfunction in older subjects with diabetes mellitus: impact on diabetes self-management and use of care services. All Wales Research into Elderly (AWARE) Study. Diabetes Res. Clin. Pract..

[32] Gonzalez JS (2008). Depression and diabetes treatment nonadherence: a meta-analysis. Diabetes Care.

[33] Hayashi K (2011). Association of cognitive dysfunction with hippocampal atrophy in elderly Japanese people with type 2 diabetes. Diabetes Res. Clin. Pract..

[34] Jongen C (2007). Automated measurement of brain and white matter lesion volume in type 2 diabetes mellitus. Diabetologia.

[35] Exalto LG, Whitmer RA, Kappele LJ, Biessels GJ (2012). An update on type 2 diabetes, vascular dementia and Alzheimer’s disease. Exp. Gerontol..

[36] Novak V (2006). Cerebral blood flow velocity and periventricular white matter hyperintensities in type 2 diabetes. Diabetes Care.

[37] Srikanth V (2011). Advanced glycation endproducts and their receptor RAGE in Alzheimer’s disease. Neurobiol. Aging.

[38] Cholerton B, Baker LD, Craft S (2011). Insulin resistance and pathological brain ageing. Diabet. Med..

[39] Last D (2007). Global and regional effects of type 2 diabetes on brain tissue volumes and cerebral vasoreactivity. Diabetes Care.

[40] Xia W (2013). Altered baseline brain activity in type 2 diabetes: a resting-state fMRI study. Psychoneuroendocrinology.

[41] Miller EK, Cohen JD (2001). An integrative theory of prefrontal cortex function. Annu. Rev. Neurosci..

[42] Kawaguchi A (2016). Insular Volume Reduction in Patients with Social Anxiety Disorder. Front. Psychiatry.

[43] Seminowicz DA (2004). Limbic-frontal circuitry in major depression: a path modeling metanalysis. Neuroimage.

[44] Price JL, Drevets WC (2010). Neurocircuitry of mood disorders. Neuropsychopharmacology.

[45] Forster S, Nunez Elizalde AO, Castle E, Bishop SJ (2015). Unraveling the anxious mind: anxiety, worry, and frontal engagement in sustained attention versus off-task processing. Cereb. Cortex.

[46] Barker H (2018). Worry is associated with inefficient functional activity and connectivity in prefrontal and cingulate cortices during emotional interference. Brain Behav..

[47] Sarinopoulos I (2010). Uncertainty during anticipation modulates neural responses to aversion in human insula and amygdala. Cereb. Cortex.

[48] Straube T, Mentzel HJ, Miltner WH (2007). Waiting for spiders: brain activation during anticipatory anxiety in spider phobics. Neuroimage.

[49] Craig AD (2002). How do you feel? Interoception: the sense of the physiological condition of the body. Nat. Rev. Neurosci..

[50] Simmons A, Strigo I, Matthews SC, Paulus MP, Stein MB (2006). Anticipation of aversive visual stimuli is associated with increased insula activation in anxiety-prone subjects. Biol. Psychiatry.

[51] Sehlmeyer C (2009). Human fear conditioning and extinction in neuroimaging: a systematic review. PLoS One.

[52] Dolcos F, McCarthy G (2006). Brain systems mediating cognitive interference by emotional distraction. J. Neurosci..

[53] Cukierman T, Gerstein HC, Williamson JD (2005). Cognitive decline and dementia in diabetes–systematic overview of prospective observational studies. Diabetologia.

[54] Ott A (1999). Diabetes mellitus and the risk of dementia: The Rotterdam Study. Neurology.

[55] Leech R, Sharp DJ (2014). The role of the posterior cingulate cortex in cognition and disease. Brain.

[56] Burwell RD, Amaral DG (1998). Cortical afferents of the perirhinal, postrhinal, and entorhinal cortices of the rat. J. Comp. Neurol..

[57] Sweatt JD (2004). Hippocampal function in cognition. Psychopharmacology.

[58] Vinckier F (2007). Hierarchical coding of letter strings in the ventral stream: dissecting the inner organization of the visual word-form system. Neuron.

[59] Kosslyn SM, Ganis G, Thompson WL (2001). Neural foundations of imagery. Nat. Rev. Neurosci..

[60] Schmahmann JD, Caplan D (2006). Cognition, emotion and the cerebellum. Brain.

[61] Rapoport M, van Reekum R, Mayberg H (2000). The role of the cerebellum in cognition and behavior: a selective review. J. Neuropsychiatry Clin. Neurosci..

[62] Daum I, Ackermann H (1995). Cerebellar contributions to cognition. Behav. Brain Res..

[63] Schmahmann JD (2019). The cerebellum and cognition. Neurosci. Lett..

[64] Beck AT, Steer RA, Ball R, Ranieri W (1996). Comparison of Beck Depression Inventories -IA and -II in psychiatric outpatients. J. Pers. Assess..

[65] Beck AT, Epstein N, Brown G, Steer RA (1988). An inventory for measuring clinical anxiety: psychometric properties. J. Consult. Clin. Psychol..

[66] Nasreddine ZS (2005). The Montreal Cognitive Assessment, MoCA: a brief screening tool for mild cognitive impairment. J. Am. Geriatr. Soc..

[67] Ashburner J (2007). A fast diffeomorphic image registration algorithm. Neuroimage.

